# Local problems; local solutions: an innovative approach to investigating and addressing causes of maternal deaths in Zambia's Copperbelt

**DOI:** 10.1186/1742-4755-8-17

**Published:** 2011-05-23

**Authors:** Mary B Hadley, Mary Tuba

**Affiliations:** 1 Copperbelt Provincial Health Office, Ndola, Zambia; 2 Ministry of Health, Vuga Road, P.O. Box 2747, Zanzibar; 3 Mwengu Social and Health Research Centre, Ndola, Zambia; 4 Current Address: Universitetet i Bergen, PO Box 7804, N-5020 Bergen, Norway

## Abstract

**Background:**

Maternal mortality in developing countries is high and international targets for reduction are unlikely to be met. Zambia's maternal mortality ratio was 591 per 100,000 live births according to survey data (2007) while routinely collected data captured only about 10% of these deaths. In one district in Zambia medical staff reviewed deaths occurring in the labour ward but no related recommendations were documented nor was there evidence of actions taken to avert further deaths. The Investigate Maternal Deaths and Act (IMDA) approach was designed to address these deficiencies and is comprised of four components; identification of maternal deaths; investigation of factors contributing to the deaths; recommendations for action drawn up by multiple stakeholders and monitoring of progress through existing systems.

**Methods:**

A pilot was conducted in one district of Zambia. Maternal deaths occurring over a period of twelve months were identified and investigated. Data was collected through in-depth interviews with family, focus group discussions and hospital records. The information was summarized and presented at eleven *data sharing *meetings to key decision makers, during which recommendations for action were drawn up. An output indicator to monitor progress was included in the routine performance assessment tool. High impact interventions were identified using frequency analysis.

**Results:**

A total of 56 maternal deaths were investigated. Poor communication, existing risk factors, a lack of resources and case management issues were the broad categories under which contributing factors were assigned. Sixty three recommendations were drawn up by key *decision-makers *of which two thirds were implemented by the end of the pilot period. Potential high impact actions were related to management of AIDS and pregnancy, human resources, referral mechanisms, birth planning at household level and availability of safe blood.

**Conclusion:**

In resource constrained settings the IMDA approach promotes the use of existing systems to reduce maternal mortality. In turn the capacity of local health officers to use data to determine, plan and implement relevant interventions that address the local factors contributing to maternal deaths is strengthened. Monitoring actions taken against the defined recommendations within the routine performance assessment ensures sustainability. Suggestions for further research are provided.

## Background

Women's health is closely linked to a country's economic productivity and growth [1]. In recognition of this association, reproductive health objectives and initiatives are included in the poverty reduction strategies in developing countries (for example: Mkuza -II, Zanzibar, 2010; Zambian Poverty Reduction Strategy, 2004; Rwandan Economic Development and Poverty Reduction Strategy, 2008). In addition, investment in the wellbeing of women facilitates realization by women themselves of their fundamental human rights. Acknowledging the importance of maternal health, the United Nations has dedicated the fifth of eight Millennium Development Goals (MDGs) to reducing maternal mortality.

Statistics show that maternal mortality is essentially a problem of developing countries. Of the 368,000 deaths associated with pregnancy and childbirth in 2009 worldwide, 99% were in developing countries. Out of these, 57% (204,000) died in sub-Saharan Africa. According to World Health Organisation (WHO) estimates women in sub-Saharan Africa have a 1:31 chance of dying as a consequence of childbirth, compared with 1:4,300 in developed regions [2].

Advancement towards meeting the 5th MDG has been acknowledged in the related progress report of 2010. A global decrease in maternal deaths of 34% from 546,000 in 1990 to 368,000 in 2009 was documented. However, in the same report, the *annual *decline of 2.3% is shown to fall well short of the 5.5% decrease required to meet the MDG target. The reduction in maternal deaths reported for sub-Saharan Africa for the same period was 26% or an annual decrease of only 1.7% [2].

Zambia, situated in sub-Saharan Africa, is one of the countries experiencing high maternal mortality. The Zambian Demographic and Health Survey (ZDHS) of 2007 [3] direct estimate of maternal mortality for the period of six years preceding the survey was 591 per 100,000 live births. In addition to survey data, the Zambian Health Management Information System (HMIS) aims to report maternal deaths from all health facilitities on a quarterly basis. However, while the ZDHS and HMIS both use the International Classification of Diseases Revision 10 (ICD 10) [4] definition of maternal deaths, until 2006, maternal deaths other than those dying of complications during delivery, were seldom captured in the HMIS.

A data audit conducted in selected hospital records in the Copperbelt Province in 2004 indicated that only deaths of women in the labour ward (11% hospital maternal deaths) were reported as maternal deaths. Deaths occuring in other wards of the hospital such as pregnancy-related deaths (39%), those due to early terminations of pregnancy (32%) and those occuring in the post partum period (18%) were not captured [5].

In one district of Zambia reviews of the reported deaths were restricted to internal medical staff discussions of labour ward deaths that took place during the routine daily clinical handover report. Reviews were not supported by documented recommendations for follow up actions.

Therefore, other than routine reproductive health activities such as antenatal screening, ad hoc initiatives to reduce maternal mortality were typically interventions conducted systematically nationwide driven by international organisations. Short re-fresher training courses in basic and emergency obstetric care for midwives is one example.

In summary, not all maternal deaths were captured in routine data and neither were causes of death well understood or documented. There was no involvement of non-medical staff in maternal death reviews. As a result interventions were not designed and implemented specifically to address the prevailing issues associated with maternal deaths in this locality.

The Investigate Maternal Deaths and Act (IMDA) approach was developed and piloted specifically to address these deficits with sustainability in mind. This model was designed by the first author, then Senior Health Adviser to the Zambian Ministry of Health, in consultation with colleagues at the Provincial and District Health Offices. A proposal for the pilot was prepared and submitted for funding. The approach consisted of four key components; identification of *all *maternal deaths according to ICD 10; investigation of *all *factors associated with the deaths involving a wide range of stakeholders; agreement on recommendations for actions required to address each identified factor by relevant decision-makers and monitoring implementation of recommended actions through the routine performance assessment process.

This article provides a summary overview of factors contributing to all maternal deaths that occurred in a district of the Copperbelt Province of Zambia in one calendar year using this IMDA approach. Selected examples of associated recommendations drawn up by the participants of the data sharing meetings are presented. Action steps associated with certain recommendations and which have been implemented by providers are provided, that illustrate the potential impact of this pilot on reduction of maternal deaths.

## Methods

The methodology for the pilot project followed the four components of the IMDA model; identification of deaths; data collection; data sharing and decision-making and; monitoring implementation of recommended activities.

### Research setting

The pilot was conducted in one district of the Copperbelt Province in Zambia between 2006 and 2007. This district had a population of 450,000 who were predominantly previous or current employees of the mining industry [6]. These inhabitants lived in low cost housing settlements on the edge of the *high cost *residential area or in rural villages located within 20 kilometres of the centre of the town. Government health services were provided by 16 health centres managed by the District Health Management Team of which six were equipped and staffed to provide maternity services. Complicated maternity cases were referred to a tertiary hospital situated in the middle of town. In 2006 the estimated number of deliveries for this district was 23,396 [6].

Ambulances were, in theory, available for referrals. In practice they were often not roadworthy or otherwise *occupied *transporting staff to and from duty shifts. Maternity services were, according to the national policy, free of charge. However, shortages of drugs and supplies resulted in the cost of certain items being passed on to the patients. These purchases were over and above the basic items a woman was expected to bring to the health facility for a normal delivery (e.g. maternity pads, gloves, baby clothes, mother's *wrap*, suture and razor blade).

At the tertiary hospital two doctors, experienced but not trained at post graduate level in obstetrics and gynaecology, were employed. These senior doctors conducted hospital ward rounds when they were available and attended to serious cases at times. Otherwise they were collected from home by the hospital transport as needed or conveyed instructions to the junior doctors by phone.

All deaths occurring within one calendar year in one DHMT catchment area made up the sample (n = 56). Family and community members, health providers including community based volunteers and others intimately involved with the case in question (traditional healers, ambulance and minibus drivers etc) were included in the data collection process.

The research team consisted of the author and principal investigator who was employed as a Senior Adviser for the Ministry of Health and the co-author as research co-ordinator. In-depth interviews were conducted by four research assistants, three female and one male, who were not related to the health sector.

### Identification of Maternal Deaths

Focal persons were identified at the hospital and the DHMT who were assigned the task of reporting all maternal deaths and passing on the details of the next of kin, with their permission, to the research coordinator. Training was provided in the use of ICD 10 definition of a maternal death. The focal persons applied the definition to all deaths occurring in women of reproductive health.

*The death of a woman while pregnant or within 42 days of termination of pregnancy, irrespective of the duration and the site of the pregnancy, from any cause related to or aggravated by the pregnancy or its management but not from accidental or incidental cause *[4].

Orientation of all midwives and traditional birth attendants on the identification of maternal deaths was provided though routine DHMT meetings to encourage the reporting of deaths outside the hospital.

### Data Collection

Zambian funerals are public affairs. Research assistants, themselves members of the local community, attended the funeral of identified women and approached the next of kin to request for an interview at their convenience. These were typically granted within seven days.

During the first and subsequent interviews, a list of persons closely connected with the woman was drawn up using a snowballing approach [7]. Interviewing continued until no new information was forthcoming. Identification, interviewing and follow ups were closely supervised by the research co-ordinator. Neighbours, *Traditional Birth Attendants*, older children, relatives, friends, employers and traditional healers were among those interviewed on a case-by-case basis. In-depth interviews were also conducted with health workers, taxi/ambulance drivers and health sector managers.

The interview guidelines were comprised of three 'grand tour' questions designed to reduce interviewer bias, building on the experience gained in a study of nurses behaviour in Bangladesh [8]. Participants were asked: *Can you tell me about this woman?: Can you tell me about her pregnancy? *and *Can you describe the events that led up to her death? *Research assistants were trained in appropriate probing techniques. At the end of each interview the research assistants allowed the respondent to give their opinion on what could be done to prevent another such death occuring.

In addition to the in-depth interviews, focus group discussions were held with traditional healers and community decision-makers, such as grandmothers and elderly married men, in order to gain a deeper understanding of issues surfacing during the interviews. Topics discussed included caesarean sections and permanent sterilisation; post mortems; use of the private section and activities of traditional healers. All interviews and discussions were tape-recorded, transcribed verbatim and typed in order to allow for either manual or electronic analyses. Health records from antenatal clinics and from health facility files and registers were reviewed and summarized.

### Data sharing

The information collected was summarized by the Principal Investigator. Demographic and socio-economic information and details of health seeking behaviour were presented to establish a profile of each woman. The place of residence, date of treatment etc. were withheld to ensure anonymity. Features of the pregnancy were then described and the remaining information shared concentrated on events leading up to the death.

Key decision-makers reviewed the available data presented during the data sharing meetings to determine the factors leading to each death. Typically four or five cases were presented and discussed at each meeting. The necessary actions required to deter another death in similar circumstances were listed and those responsible for their implementation agreed upon them. A visiting obstetrician and in-house clinicians decided when a diagnosis was accurate or a particular treatment appropriate in reference to treatment protocols.

### Analysis

At the end of the pilot project, when all deaths had been reported, investigated and reviewed, an analysis of the main contributing factors was conducted. Key themes were identified, which are presented in this article. The frequency of factors within each theme and sub-heading was calculated. While the sample size was too small to establish statistical significance the frequency data was used to identify tendencies for themes to overlap using Microsoft Excel pivot tables. Finally the proportion of recommendations implemented was calculated.

### Monitoring and sustainability

During the IMDA pilot a simultaneous exercise was conducted to revise the health sector performance assessment tools. Sustainability of the IMDA approach was ensured through the inclusion of a minimum standard indicator in the revised routine performance assessment tool. The indicator is: *number of recommendations implemented *divided by *the number of recommendations drawn up during maternal death reviews*. Performance self-assessments are conducted bi-annually by each facility and District Health Office and reviewed by the administrative level above. Technical support to address the deficits is provided as agreed during the Performance Assessment discussions, following the national health sector procedures. Inclusion of IMDA monitoring in the national health system was an important component of the model.

### Ethical considerations

Ethical clearance was provided through the Tropical Disease Research Centre, Ndola, Zambia. The Ministry of Health at Central, Provincial and District level gave permission for the research team to access maternal health records and health providers. Participants were explained the purpose of the study and written consent was obtained before interviews commenced. Transcripts were coded to prohibit identification of the participant to those outside the immediate study team.

The IMDA pilot was funded through Irish Aid, Lusaka Office.

## Results

A total of 56 maternal deaths were identified of which 2 occurred at home, 1 in the health centre and 53 at the tertiary hospital. The average age of the women at time of death was 26.7 (range 16-40). Mean number of reported previous pregancies was 3.2 (range 0-12) and living children 2 (range 0-11). A breakdown of the age groups is presented in Table 1 compared with national age groups of maternal deaths. National data includes deaths from rural areas while the IMDA study was conducted in an urban setting which may account for the younger age groups showing higher death rates. Age specific data for all deliveries are nto available and demographic and previous obstetric data is not available for seven maternal death cases in the IMDA pilot.

**Table 1:** Age comparison of maternal deaths between ZDHS 2007 and IMDA pilot

	National (2001-7 ZDHS)		IMDA (2006)	
**Age Group**	**Annual average**	**% maternal deaths**	**IMDA (2006)**	**% maternal deaths**

15-19	4.8	5%	6	12%

20-24	13.7	13%	8	16%

25-29	23.4	22%	14	29%

30-34	32.2	30%	15	31%

35-39	20.9	20%	5	10%

40-44	7.3	7%	1	2%

45-49	3.4	3%	0	0%

**15-49**	**105.8**	**100%**	**49**	**100%**

no date of birth			7	

**Total**	**105.8**		**56**	

The death of a woman while pregnant or within 42 days of termination of pregnancy, irrespective of the duration and the site of the pregnancy, from any cause related to or aggravated by the pregnancy or its management but not from accidental or incidental cause [4]

### Factors contributing to maternal deaths

**Figure 1:**
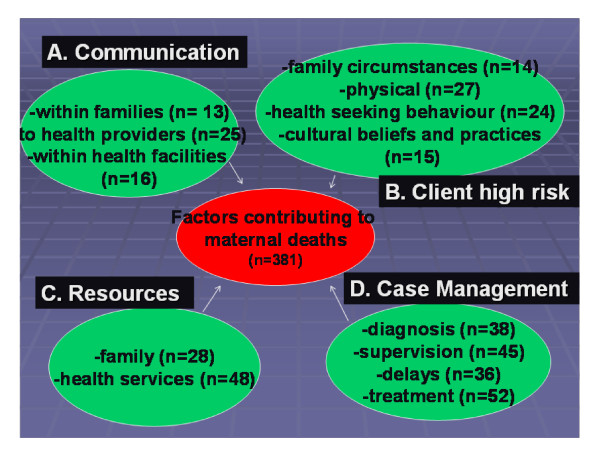
**Factors contributing to maternal deaths in Copperbelt Zambia by theme and frequency, 2006 (IMDA pilot)**.

Several factors were associated with a single death (mean = 6.8). The major themes that emerged during the final analysis of these factors were; poor communication; client high risk factors; lack of resources and issues related to case management. The frequency of factors and sub headings for each theme are presented in Figure 1.

In the following section testimonies from relatives and health providers provide a more in-depth understanding of the challenges facing this district in relation to maternal health. Recommended actions that were drawn up during the data sharing meetings were determined on the basis of this information on a case-by-case basis.

#### A. Communication

Poor communication within families, between families and health providers and within the players in the health system resulted in wrong or delayed decisions being made. There was, in particular, an unwillingness to disclose pregnancy status, abortions or HIV status, even to family members. The case of a 16 year old schoolgirl, Mwaka (alias) illustrates this reluctance. Mwaka had a relationship with a married soldier serving in a military camp situated near the school she attended. She stayed with her aunt during term time. Mwaka secretly aborted a pregnancy from this relationship, using a foreign body inserted through the vagina. This she only disclosed in her dying breaths. Her twin sister, who was also hiding a pregnancy at the time, gave this account...

"She (my sister) removed it (the blood- soaked cloth) and took it behind the house. So as mummy was cleaning outside she saw the blood. That is when she (the mother) came and confronted her but still she (my sister) refused to tell her anything. So mummy took her to the clinic."

Her sister also commented that Mwaka would not have known how to abort a pregnancy, '*someone must have helped her'*. During an interview her mother indicated that she would have offered to take care of the child while her daughter continued her education had she been aware that her daughter was pregnant- her own children were grown up. The cause of death as stated on Mwaka's death certificate was *septic abortion*. Poor communication within families was identified in 33% of HIV deaths and 78% of self induced abortions, as in Mwaka's case.

Reluctance of patients and their family members to share vital information with health providers interfered with the diagnosis of seriously ill patients. Cultural beliefs and norms also played a role (see below). The use of traditional herbal remedies is commonplace in Zambia and results in a display of signs and symptoms unexplained in medical textbooks. There was a reluctance to disclose use of herbal medicines to scornful health providers, resulting in considerable *guesswork *by clinicians in drawing up a diagnosis.

Poor communication between health facilities also resulted in delayed treatment. The tertiary hospital attended to all critically ill patients as they arrived on the maternity ward, without forewarning, from the referring health centre. This practice reduced the preparation time for medical consultation and life-saving surgical interventions at the tertiary hospital.

Similarly, within the tertiary hospital communication was less than optimal. Exchange of information between the laboratory and the ward, as well as between senior staff left the physician managing seriously ill women *blindfolded*. This was evident from the absence of feedback relating to the requested laboratory tests, and other specialists' expert opinions as illustrated in the summary of one patient's hospital notes Table 2). It demonstrates that on numerous occasions' laboratory tests, specialist (surgical) opinions and HIV tests were requested by the attending physician. Limited family and health facility resources, however, may also have influenced the availability of laboratory tests results (see section resources).

**Table 2:** A summary of entries into a critically ill patient's chart in the post partum period

Day one-15.15
Delivered 7 days ago
Chronically ill looking, pallor, Chest- AE down bilaterally, dull on percussion, S1, S2 normal, pus aspirated on both sides
R/O (sic.Rule out) RVD (sic. HIV)
CXR, FBC,ESR,LFT,ICD,VCT,ATT
Day 2
VCT requested- Cotrimoxazole given
Day 3
Follow up lab tests, VCT
Day 4
No entry
Day 5
Follow up lab tests, VCT
Day 6
Follow up lab tests, VCT, consider ICD, Surgeon to see
Day 7
Repeat lab tests, VCT, surgeon to see, trace lab results
Day 8
Repeat lab tests, surgeon to see, trace lab results
Day 9
Repeat CXR, follow up on lab results, surgeon to see
Day 10
R/O RVD (sic HIV), follow up lab results, VCT, repeat U/E, LFT
Day 11
FBC/ESR, Urgent HB, CXR, Surgical consultation, Lasix, CST, O2 therapy
Day 12
Surgical consultation, CXR, Trace lab results
Day 13
VCT
Day 14
VCT, RPR, surgical consultation
Day 15
VCT, RPR
Day 16
Add slow K, Prednisolone
Day 17
(Not seen in am)
Certified dead at 14.26

#### B. Client High Risk

**Figure 2:**
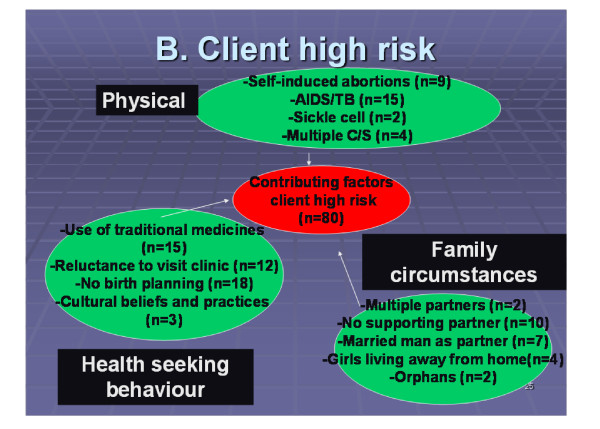
**Breakdown of pre-existing client risk factors identified during IMDA pilot, Zambia 2006**.

Physical conditions, health seeking behaviour and family circumstances (n = 80) were identified as pre-existing circumstances/conditions that complicated the provision of care during pregnancy, childbirth and the post partum period in 38 of the maternal deaths. The sub themes under each of these client risk factors are presented in Figure 2.

Pre-disposing medical conditions such as sickle cell anaemia, HIV and a history of previous multiple caesarean sections overstretched the ability of health facilities to provide the care required for safe childbearing. Women with HIV/AIDS, already in a compromised immune state, were particularly vulnerable. In addition, in the absence of lawful, safe services for termination of unwanted pregnancies women, like Mwaka, presented with complications of self-induced abortions.

Family and community interviews revealed traditional practices that complicated standard pre and post-natal management by health providers. Reluctance to use family planning to avert unintended pregnancies and to utilise services at the local health facilities were predisposing factors contributing to maternal deaths; it goes without saying that a woman who is protected from pregnancy through family planning cannot be added to the statistics of maternal mortality. One third of deaths identified in the IMDA pilot were reported by family members and friends as being *unplanned*.

The widespread use of alternative traditional medicines was not without complications. In particular, a locally available herbal substitute for oxytocin was used to speed up labour by intensifying uterine contractions. Even trained midwives use this natural product during their deliveries. Linked to the suspicion that a prolonged labour signified infidelity in the marriage and could lead to death (locally known as *inchila*), labour of short duration was preferred. The same belief was also responsible for delay in seeking care on onset of labour. Traditional family norms also influenced a pregnant woman's decision to seek health care. Women needed the financial, logistical and moral support from their male partners to receive care in health facilities as one neighbour of a woman who had died as a result of post partum sepsis illustrated.

*Her husband used to refuse to take her to the clinic ....When she was very sick she was taken by her brother on the bicycle. She delivered alone. The cord was around the neck *[of the newborn baby]. *So she asked her older child to call the neighbour who cut the cord*.

#### C. Resources

Delays in seeking and managing complications of childbearing were related to a scarcity of family resources in half of all deaths and shortages of supplies within the health facility in 86% of deaths.

Private ownership of transport, even bicycles, was limited to the privileged few in this district of Zambia. Walking and travel in locally-run and overcrowded minibuses were the most common forms of transport. Securing transport took precious time and was identified as a factor contributing in a quarter of the cases. In rural parts of the district, as well as some high population density areas, wheelbarrows were considered the most comfortable non-motorised transport for seriously ill women. While taxis were used in emergencies, not all drivers agreed to take a woman who was bleeding in their vehicle. Once at the health centre the difficulty in securing an ambulance resulted in a high reliance on taxis for referral purposes. In the surrounding villages there was no motorised transport of any description. Selena (alias) died in a village four hours' walk from the nearest health facility. Her husband described what happened...

*At around 03.00 hours in the night she told me that she felt a pain in her back. Then I said 'Here there are no vehicles, what will happen'? We stayed and slept again till 06.00 that's when she delivered a baby girl. It was not long afterwards, she just said don't lift me; let me lie down a bit. When she did that, she even died. It was around 07.00 hours*.

The cause of death on Selena's death certificate was post-partum haemorrhage.

Stock-outs of drugs, supplies and even blood units for transfusion resulted in the cost of maternity care being passed onto the woman and her family. Twenty-six out of 28 deaths with contributing factors related to limited family resources (98%) also identified health facility resources as being an issue. Prescriptions were given for medicines and supplies, which in turn were purchased in local pharmacies and drug stalls. These purchases required cash. Delays in sourcing cash in an emergency situation were experienced by half of the families interviewed. This was the testimony of a husband whose wife died in the hospital because of a septic abortion.

*...they wrote the prescription on the day she died. Ok, what happened was...my mother was at the bedside, so when I arrived I found they *(doctors) *had already written the prescription. So as I was leaving again *(to purchase the prescribed medicines from the private pharmacy) *I was called that she had died*.

At the tertiary hospital the absence of a fully qualified obstetrician had several consequences. Firstly, women did not have access to the specialised care required for a serious complication. Secondly, junior doctors diagnosed and managed cases beyond their capacity. They learnt by trial and error. Errors were translated into death in extreme situations and recognised as a contributing factor in 80% of cases investigated.

Blood units for transfusion were collected by the blood bank staff during mobile outreach collection sessions, typically at schools and colleges. After screening, the blood was distributed according to requests and availability. Shortages of blood during school holidays were commonplace and contributed to 27% deaths. Even for cases where it could have been predicted that a blood transfusion would be required, blood units were not available. The husband of one of the two sickle cell patients who died in childbirth in the hospital during the pilot gave his opinion...

Interviewer: Do you think the death of your wife would have been prevented?

*Informant: Yes, anyway it would have been prevented if the doctors had taken measures to find the blood to replace the blood maybe it would have helped her. Because she had lost blood, you see*.

#### D. Case management

Poor case management was exacerbated by the absence of skilled personnel for individual case management and for supervision of the junior doctors. A reluctance to take the advice of very experienced midwives on the ward further aggravated the situation.

There was evidence of 'norms' within the hospital that interfered with diagnosis and treatment. During the data sharing meetings clinicians agreed that over two thirds (67%) of cases did not have accurate diagnosis. Although during a focus group discussion community members expressed the need for post mortem examinations to establish cause of death medical staff were reluctant to organise such investigations, using cultural norms and beliefs as reasons. However, traditionally a woman in Zambia is not buried with an unborn child, meaning that families paid the hospital pathologist to surgically remove a foetus in the morgue. The opportunity to conduct an investigation during this procedure was missed.

Malaria is typically used as a diagnosis when there is an unknown cause of illness in this previously high endemic malaria region. However, with the introduction of effective preventative and treatment initiatives, there was little evidence that the burden of malaria posed a significant risk in this district at the time of the pilot study. Malaria diagnosis was, however, recorded as a diagnosis unsupported by clinical signs and symptoms or laboratory confirmation. The result being that the real cause of ill health was not investigated in seven women who subsequently died.

There were delays in giving treatment, especially operations and antibiotics. The reasons for this were interlinked. Poor case management, lack of supplies at the hospital, reluctance to perform lumbar punctures and absence of readily available cash at the home all played a part. A summary of the frequencies within each theme of both contributing factors and the number of cases affected is presented in Table 3.

**Table 3:** Summary of frequencies of contributing factors and cases within key themes

Themes	Cases (n = 56)	Contributing factors (n = 381)
A. Communication	38	54

B. Client risk factors	38	80

C. Resources	50	76

D. Case Management	54	171

#### Recommendations

A total of eleven data sharing meetings were conducted, chaired by the Provincial Health Director. Participants included provincial and district level officers including the responsible focal persons for reproductive health, hospital and health centre staff, traditional birth attendants, visiting obstetricians and representatives from the blood transfusion centre. The exact composition of the groups depended on the key issues that had emerged during data collection. The factors contributing to the deaths were discussed during the meetings where a total of 68 recommendations were drawn up.

The level within the health system at which the recommendations were to be implemented varied. Those requiring high level interventions or significant additional resources required action to be taken initially at national level. However the results of the study provided a powerful lobbying tool for the senior district and provincial officials, for example in articulating the need for obstetricians at the tertiary hospital. The distribution between the different levels was: District Health Office 36%; Provincial Health Office 9%; research institute 3%, Ministry of Education 1% (n = 1) and the tertiary hospital 54% (n = 34).

In addition to actions requiring central level intervention activities to improve management, strengthen monitoring of existing procedures and allocation or re-allocation of resources were agreed upon. Examples of actions taken against the recommendations to address selected problems identified at the data sharing meetings are presented in Table 4. The number of cases that this action was recommended for, to avert maternal deaths occurring due to the same circumstances, are included.

**Table 4:** Examples of factors contributing to maternal deaths and recommendations for actions to be taken

Category	Problem identified	Recommendation	Action taken (number of cases affected)
Case management	-Junior doctors working unsupervised -Complicated cases undiagnosed -Incorrect case management -Deaths during caesarean section	Obstetricians required for tertiary hospital	Obstetrician allocated to the hospital (recommended for **52 **cases)
	
	-Deaths during operation for routine Caesarean sections in women who had already multiple (up to 5) previous Caesarean sections	Counselling for bilateral tubal ligation at antenatal clinics for all clients having three previous Caesarean sections	Midwives sensitised on the need for counselling for women who already had three caesarean sections to obtain consent for sterilisation for the next delivery during routine antenatal clinics Counselling introduced in all health centres (recommended for **4 **cases)
	
	Patients die undiagnosed	Conduct post mortems when maternal death cause is unknown	Post mortem was conducted when cause of death was not clearly identified (recommended for **38 **cases)

Communication	Short time for preparing referred patients for emergency operations	Monitor the preparation of women who are referred from health centres, keep chart in labour ward DHMT to orientate midwives in safe referrals during midwives routine monthly meeting DHMT Reproductive Health Officer to collect monitoring sheet and provide feedback and support to health centres	A monitoring chart relating to communication between a referring clinic and the labour ward of the tertiary hospital regarding referral cases introduced and monitored by District reproductive health officer (recommended for **54 **cases)

Resources	No antibiotics available for treatment of post partum or post abortion sepsis	Maintain a supply in ICU for maternal cases	A stock of antibiotics established in Intensive Care Unit (ICU) for use for septic abortion and post partum sepsis (recommended for **28 **cases)
	
	-Blood units not always available on request especially during school holidays -No documented system of ordering and receiving blood	Establish a Blood transfusion committee to discuss blood collection and distribution Diversify the register of blood donors Review and document ordering system.	Campaigns to collect blood from regular donors resulted in adequate stocks -Regular meetings with Zambia Blood Transfusion Services and its stakeholders held -Ordering system put in place to document requests and receipts of blood units. *There were no shortages of blood for transfusion in subsequent years* (recommended for **15 **cases)
	
	-Clients do not have readily available cash for transport to attend health centre for delivery. -Patients do not have supplies required by health centre resulting in home deliveries.	Advice and support for birth planning for all pregnant women - design strategies to include male partners. -Re-introduce safe birthing kits for pregnant women at antenatal clinics	-Birth planning sessions, both group and individual, introduced at all antenatal clinics -Safe birthing kits not re-introduced at the end of pilot (recommended for **28 **cases)

Client High risk	Deaths of women who were HIV positive or suspected AIDS patients	Full ART for all pregnant women	Regime for HIV positive pregnant women changed from Niverapine only to full ART (recommended for **15 **cases)

	Septic abortions due to self induced abortions	-Extend access to family planning services to women aged >35 and under 20 -Provide Post Abortion Care services at hospital	Midwives ortiented -Post abortion care services introduced (recommended for **9 **cases)

#### Recommendations

A total of eleven data sharing meetings were conducted, chaired by the Provincial Health Director. Participants included provincial and district level officers including the responsible focal persons for reproductive health, hospital and health centre staff, traditional birth attendants, visiting obstetricians and representatives from the blood transfusion centre. The exact composition of the groups depended on the key issues that had emerged during data collection. The factors contributing to the deaths were discussed during the meetings where a total of 68 recommendations were drawn up.

The level within the health system at which the recommendations were to be implemented varied. Those requiring high level interventions or significant additional resources required action to be taken initially at national level. However the results of the study provided a powerful lobbying tool for the senior district and provincial officials, for example in articulating the need for obstetricians at the tertiary hospital. The distribution between the different levels was: District Health Office 36%; Provincial Health Office 9%; research institute 3%, Ministry of Education 1% (n = 1) and the tertiary hospital 54% (n = 34).

In addition to actions requiring central level intervention activities to improve management, strengthen monitoring of existing procedures and allocation or re-allocation of resources were agreed upon. Examples of actions taken against the recommendations to address selected problems identified at the data sharing meetings are presented in Table 4. The number of cases that this action was recommended for, to avert maternal deaths occurring due to the same circumstances, are included.

#### Monitoring and sustainability

**Figure 3:**
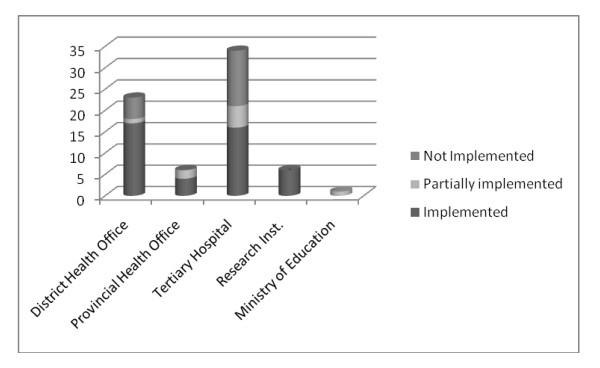
**Progress of implementation of recommendations at end of IMDA pilot**.

At each data sharing meeting progress against the recommendations was reviewed. At the time of concluding the pilot 61% of recommendations had been implemented, 12% partially implemented and 27% not implemented. Figure 3 provides a breakdown of the status at the year end.

Performance assessments which included the minimum standard indicator related to implementation of necessary action in maternal death reviews were conducted every six months from 2006.

## Discussion

The increased proportion of maternal deaths captured using this approach allowed for investigation of self-induced abortions and complications in the prenatal and post partum periods. The deaths occurring at home were reported by the Traditional Birth Attendants who had been made aware of the ICD 10 definition of a maternal death. While community members held the view that home deaths are rare events a routine investigation of all deaths occurring in the community through vital registration should be considered in further use of the IMDA approach and is a limitation of this pilot. A second limitation of the IMDA pilot was the reliance on a woman dying to address maternal death risk factors. The inclusion of 'near miss' cases [9] should be considered, depending on number of deaths and capacity for investigation and follow up.

The summary of contributing factors illustrates that no deaths were as a result of just one cause or reason, most had several. The use of 'grand tour' questions and inclusion of general information about the woman resulted in a comprehensive overview of the situation. This contrasts with the World Health Organisation 'Beyond the Numbers' [10] approach to maternal death reviews. This approach uses semi-structured questionnaires culminating in a primary and a limited number of secondary causes of death being attributed to each death. This allows for comparison between countries and within countries over time of biomedical causes of death. The IMDA approach, on the other hand, is primarily concerned with identifying all contributing factors as a basis for determining interventions at a local level. The cause of death on Selena's death certificate was post-partum haemorrhage (PPH). The contributing factors were related to her physical location, lack of resources to travel to a health facility and the degree of preparedness for the eminent birth. Cultural factors including the knowledge of her husband of pre-requisites for safe motherhood also played a role. Interventions for PPH are typically improving midwives' delivery skills, use of misoprostal and the availability of safe blood. None of these interventions would have averted Selena's death unless other measures were taken to ensure she had access to these services. So while the ICD 10 definition was helpful in identifying more maternal deaths than previously had been, the biomedical cause of each death according to the same classification document was attributed less importance using the IMDA approach.

The recommendations were drawn up by the very people who were responsible for their implementation. As a result translation of recommendations into actions was high, at 67%. However, due to the sensitive nature of some emerging issues such as unplanned (particularly teenage) pregnancies and strong religious convictions, direct recommendations for a change in the abortion law and introducing family planning advice as well as offering commodities to teenagers were not included formally in the list of interventions. The forum, however, provided a unique opportunity to air controversial issues, otherwise never formally discussed.

In 2006 the HIV prevalence in Copperbelt Province was 24% of the adult population. The scale of the contribution of AIDS to maternal deaths was recognised as the pilot progressed. A change in the drug regime from Niverapine only to full ART for eligible HIV positive pregnant women was introduced as a response.

In the whole year, no recommendation was made for re-fresher training in delivery skills for midwives other than the use of magnesium sulphate for eclampsia patients. Inadequate skills for assisting deliveries was not identified as contributing to any of the 56 deaths. According to the study results, the refresher training that was conducted for midwives would be unlikely to have a major impact on reducing the high maternal mortality in this district. This is important to note since cost effective and relevant interventions were at the heart of the design of the IMDA approach.

## Conclusion and Recommendations

In resource constrained settings the IMDA approach promotes the use of existing systems to reduce maternal mortality. In turn the capacity of local health officers to use data to determine, plan and implement relevant interventions that address the local factors that contribute to maternal deaths is strengthened. Monitoring actions taken against the defined recommendations within the routine performance assessment ensures sustainability.

The authors recommend the addition of a community-based identification system using the vital registration system and to include all deaths of women of reproductive age to improve the comprehensiveness of the identification process.

Furthermore, a comparison of the factors identified using the three 'grand tour' question approach and those identified using the WHO Beyond the Numbers, semi-structured tool is recommended. Such a comparison would assist in determining the most accurate data collection technique for the determination of appropriate and cost effective interventions to reduce maternal mortality.

Finally, a follow-up study is recommended to evaluate the impact of this study and its recommendations through analysis of the number of women dying in subsequent years dying due to the same identified factors. A comparison with a neighbouring district, as control, would strengthen this evaluation.

## Competing interests

The author declares that they have no competing interests.

## Authors' contributions

MB conceived and designed the IMDA model, participated in data collection, conducted the qualitative data analysis including triangulation of data, drafted the manuscript and performed the frequency analysis. MT managed and actively participated in the data collection and participated in the qualitative data analysis. Both authors read and approved the final manuscript.
